# Neonatally induced mild diabetes: influence on development, behavior and reproductive function of female Wistar rats

**DOI:** 10.1186/1758-5996-5-61

**Published:** 2013-10-16

**Authors:** Ana Carolina Inhasz Kiss, Barbara Woodside, Yuri Karen Sinzato, Maria Martha Bernardi, Wilma De Grava Kempinas, Janete Aparecida Anselmo-Franci, Débora Cristina Damasceno

**Affiliations:** 1 Department of Physiology, Botucatu Biosciences Institute, São Paulo State University, Distrito de Rubião Júnior s/n, 18618-970, Botucatu, São Paulo, Brazil; 2 Laboratory of Experimental Research of Gynecology and Obstetrics, Department of Gynecology and Obstetrics, Botucatu Medical School, São Paulo State University (Unesp), Distrito de Rubião Júnior s/n, 18618-970, Botucatu, São Paulo, Brazil; 3 Center for Studies in Behavioral Neurobiology, Psychology Department, Concordia University, 7141 Sherbrooke St. W., Montreal H4B 1R6 QC, Canada; 4 Mathematics, Computation and Cognition Center, Federal University of ABC, Av. dos Estados, 5001, 09210-580, Santo André, São Paulo, Brazil; 5 Graduate Program of Environmental and Experimental Pathology and Graduate Program of Dentistry, Paulista University, Rua Dr. Bacelar, 1212, São Paulo, São Paulo, Brazil; 6 Department of Morphology, Botucatu Biosciences Institute, São Paulo State University, Distrito de Rubião Júnior s/n, Botucatu 18618-970 São Paulo, Brazil; 7 Laboratório de Neuroendocrinologia da Repdrodução, Departamento de Morfologia, Estomatologia e Fisiologia, Faculdade de Odontologia de Ribeirão Preto, São Paulo University, Av. do Café s/n, 14040-904, Ribeirão Preto, São Paulo, Brazil

**Keywords:** Mild diabetes, Streptozotocin, Rat, Development, Behavior, Reproductive function

## Abstract

**Background:**

Neonatal STZ treatment induces a state of mild hyperglycemia in adult rats that disrupts metabolism and maternal/fetal interactions. The aim of this study was investigate the effect of neonatal STZ treatment on the physical development, behavior, and reproductive function of female Wistar rats from infancy to adulthood.

**Methods:**

At birth, litters were assigned either to a Control (subcutaneous (s.c.) citrate buffer, n = 10) or STZ group, (streptozotocin (STZ) - 100 mg/kg-sc, n = 6). Blood glucose levels were measured on postnatal days (PND) 35, 84 and 120. In Experiment 1 body weight, length and the appearance of developmental milestones such as eye and vaginal opening were monitored. To assess the relative contribution of the initial and long term effects of STZ treatment this group was subdivided based on blood glucose levels recorded on PND 120: STZ hyperglycemic (between 120 and 300 mg/dl) and STZ normoglycemic (under 120 mg/dl). Behavioral activity was assessed in an open field on PND 21 and 75. In Experiment 2 estrous cyclicity, sexual behavior and circulating gonadotropin, ovarian steroid, and insulin levels were compared between control and STZ-hyperglycemic rats. In all measures the litter was the experimental unit. Parametric data were analyzed using one-way or, where appropriate, two-way ANOVA and significant effects were investigated using Tukey’s post hoc test. Fisher’s exact test was employed when data did not satisfy the assumption of normality e.g. presence of urine and fecal boli on the open field between groups. Statistical significance was set at p < 0.05 for all data.

**Results:**

As expected neonatal STZ treatment caused hyperglycemia and hypoinsulinemia in adulthood. STZ-treated pups also showed a temporary reduction in growth rate that probably reflected the early loss of circulating insulin. Hyperglycemic rats also exhibited a reduction in locomotor and exploratory behavior in the open field. Mild hyperglycemia did not impair gonadotropin levels or estrous cylicity but ovarian steroid concentrations were altered.

**Conclusions:**

In female Wistar rats, neonatal STZ treatment impairs growth in infancy and results in mild hyperglycemia/hypoinsulinemia in adulthood that is associated with changes in the response to a novel environment and altered ovarian steroid hormone levels.

## Background

Several methods have been used to reproduce in laboratory animals the hyperglycemic state of diabetic patients among these is the administration of beta-cytotoxic chemical agents, such as streptozotocin (STZ). STZ damages the pancreatic beta-cell initially causing release of insulin leading to a transient hyperinsulinemia/hypoglycemia and then resulting in longterm hypoinsulinemia/hyperglycemia [1].

Depending on the route, dose, age, and strain of animals, STZ may induce severe or mild diabetes. When rats are rendered diabetic using STZ administration during adult life they present severe diabetes, with blood glucose levels above 300 mg/dl and often exceeding 500 mg/dl. Glycemic levels within this range are rare among the human diabetic population. As a consequence, experimental manipulations that result in animals presenting mild hyperglycemia (between 120 and 300 mg/dL) have been developed (see [2] for a review). In contrast to the permanent effects of STZ seen following treatment of adult rats, neonatal administration of this drug produces only a transient effect on insulin levels because there is a spontaneous recovery of the beta-cells over the first 2 weeks of life, resulting in normoglycemia until at least 6–8 weeks of life. However, beta-cell regeneration is often incomplete leading to reduced beta-cell mass [1], impaired glucose tolerance and reduced plasma insulin in adulthood [3].

Although a number of studies have shown the consequences of neonatally STZ induced mild diabetes on several metabolic parameters [1,3-10] little is known about how this neonatal insult might affect physical development or behavior in infancy and adult life. In addition, although behavioral changes in the open field [11-15] and compromised reproductive function [16,17], including impaired sexual behavior [18-20], have been reported in animals presenting severe diabetes, little is known about the impact of neonatal STZ injection and the ensuing mild hyperglycemia on these parameters. Thus, the aim of the present study was to evaluate the effect of neonatally induced mild diabetes on physical development, behavior, and reproductive function, of female Wistar rats from infancy to adulthood.

## Methods

### Subjects

The female rats used in these studies were the offspring of male and female Wistar rats obtained from São Paulo State University (Unesp), Botucatu, São Paulo State, Brazil. They were maintained in an experimental room under controlled conditions of temperature (22 ± 2°C), humidity (50 ± 10%), and a 12 h light/dark cycle. All experimental procedures were approved by the local Committee of Ethics in Animal Experimentation (Number 665), which assures adherence to the standards established by the NIH Guide for the Care and Use of Laboratory Animals.

### Experimental procedure

**Figure 1:**
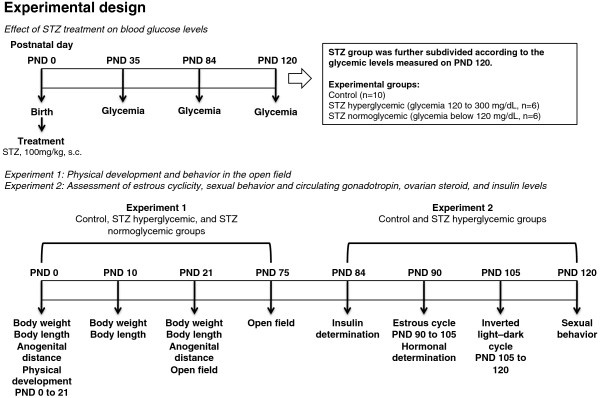
Experimental design.

All experimental procedures are summarized on Figure 1. Rats used in this study were the offspring of 16 females that were mated at 90 days of age. The morning on which sperm were found in the vaginal smear was designated pregnancy day 0. Around pregnancy day 22 the rats delivered naturally. Litters of these females were randomly assigned to one of two groups. Control litters (n = 10) in which pups received subcutaneous injections of citrate buffer (0.1 M, pH 4.5) on first day of life (postnatal day 0 - PND 0); and STZ litters (n = 6) in which pups received a subcutaneous injection of 100 mg/kg streptozotocin (STZ, SIGMA Chemical Company, St. Louis, Millstone) [10,21] on PND 0. STZ or vehicle was carefully injected in the pups to minimize leakage of solution. This experimental model has been used successfully in several studies to induce mild hyperglycemia [3,22-26]. The number of pups per litter was standardized at 8 females and pups remained with their mothers until weaning. The mortality rate of the STZ group was 8%.

Two cohorts of rats underwent this treatment. Females in the first cohort were used in experiment 1 to evaluate the effects of neonatal STZ treatment on physical development, blood glucose levels and open field behavior. In Experiment 2 the effect of this manipulation on estrous cyclicity, sexual behavior, and circulating levels of insulin and reproductive hormones were assessed in females from a separate cohort.

### Effects of STZ treatment on blood glucose levels

On postnatal day (PND) 35, 84 and 120, blood samples were obtained from a tail nick for glycemic determinations (glucose oxidase) using a commercial glucometer (*One Touch Ultra*, Johnson & Johnson®) and values were expressed in milligrams per deciliter (mg/dl). All animals were tested during the light phase of the cycle, 4 to 5 hours after lights on. Rats were not fasted for glycemic determinations.

### Experiment 1: Physical development and behavior in the open field

This STZ treatment creates a range of damage to beta cells, leading to a variable range of insulin insufficiency in adulthood [2]. Thus, for these measures the STZ group was further subdivided according to the glycemic levels measured on PND 120: STZ hyperglycemic (18 offspring from the 6 STZ-treated litters), having glycemic levels between 120 and 300 mg/dl; and STZ normoglycemic littermates (14 offspring from the 6 STZ treated litters), that had received STZ solution but had blood glucose levels lower than 120 mg/dL in adult life. Where more than one pup from a litter was tested on a measure the scores were averaged across pups so that the litter remained the experimental unit.

### Physical development

Pups from each litter were observed daily from PND 1 to 20 in order to evaluate physical development according to the methodology proposed by Smart & Dobbing [27]. Eye opening, hair appearance, pinna unfolding, incisor eruption, and vaginal opening were observed until 100% of the pups in the litter reached that developmental landmark.

Body weight and naso-anal body length were measured on PND 0, 10 and 21. Body weight gain and change in body length between PND 0 to 10 and 10 to 21 were calculated. Calipers were used on PND 10 and 21 to measure anogenital distance: the length (millimeters) from the anal to the genital opening. Because anogenital distance can be influenced by body length an anogenital index was calculated as anogenital distance/body length.

### Open field behavior

Two open fields similar to that described by Broadhurst [28] were used in this study. To evaluate the behavior of rats on PND 21, the open field consisted of a wooden circular arena with a diameter and height of 40 cm. An arena with diameter 100 cm and height 40 cm was used for adult rats on PND 75. The floor of both arenas was divided into 25 approximately equal sections. All testing was carried out in a quiet, diffusely lit room between 2 and 5 p.m during the light phase of the day/night cycle. To minimize possible influences of circadian rhythmicity on rat behavior in the open field, testing of control and experimental rats was interspersed. The apparatus was cleaned with a 5% alcohol solution between each test.

Behavior in the open field was used for assessment of general activity on PND 21 and 75. Each animal was placed in the arena and the following parameters were observed for five minutes:

● Ambulation: number of sections entered with all 4 paws;

● Rearing: number of times the animal stood on its hind limbs;

● Immobility: time in seconds the animal did not show motor activity and its head, trunk and limbs remained static;

● Self-grooming: time in seconds the animal performed this activity.

Two observers scored the behavior using hand-operated counters and stopwatches. The presence of urine, and fecal boli were also recorded and analyzed.

### Experiment 2: Assessment of estrous cyclicity, sexual behavior and circulating gonadotropin, ovarian steroid, and insulin levels

Only rats from Control (blood glucose levels lower than 120 mg/dL) and STZ hyperglycemic (glycemic levels between 120 and 300 mg/dL) groups were used in this study. 2 to 3 female rats from each litter were tested on each parameter and the scores/measures averaged within a litter.

### Estrous cycle duration

Stage of the estrous cycle was assessed by light microscopic examination of cells obtained from vaginal smears collected every morning over a period of 15 days (PND 90 to PND 105). Estrous cycle phase was estimated based on cell type and relative frequency [29,30]. Estrous cycle duration was calculated based on the number of days between one estrous phase to the next.

### Determination of circulating gonadatropin and ovarian steroid concentrations

During the estrous phase of the natural estrous cycle, blood samples were collected from the caudal vein into heparin-coated syringes, at 09:00 a.m. Immediately after collection, blood samples were centrifuged (2500 rpm for 20 min at 2C) and the plasma frozen at -20C until the assays were performed. Plasma LH and FSH were determined by double antibody radioimmunoassay using specific kits provided by National Hormone and Peptide Program (National Institutes of Diseases Digestive and Kidney, USA). The concentration of plasma progesterone and beta-estradiol was determined by double antibody radioimmunoassay using specific kits provided by MAIA (BioChem ImmunoSystems, Itália S.P.A). All samples were measured in duplicate and, when needed, at different dilutions. All samples from the same experiment were measured in the same assay.

### Sexual behavior

Rats were maintained under controlled temperature conditions on a reversed 12-h light–dark cycle (lights on at 10 p.m.) for 15 days prior to the tests, with ad libitum access to food and water. Around PND120, the mating tests were performed according to the methodology described by Felício and Nasello [31]. Sexual behavior was assessed in cycling rats 3 to 4 hours after a proestrous smear was observed. Sexually experienced males were used in these tests which lasted until ten mounts had been observed [32]. Results were expressed as the lordosis quotient (LQ, number of lordosis/ ten mounts X100) [33]. All rats were tested only once.

### Insulin determination and glycemia

On PND 84 blood samples were obtained for plasma insulin determination by ELISA (Mercodia®) and values were expressed as milligrams per liter (mg/L). On PND 120, blood samples were obtained from a tail nick for glycemic determinations (glucose oxidase) by a usual glucometer, and values were expressed in milligrams per deciliter (mg/dL). All animals were tested during the light phase of the cycle, 4 to 5 hours after lights on. Rats were not fasted for glycemic determinations. The mild diabetic condition was confirmed with blood glucose concentration ranging between 120–300 mg/dL in adult rats [34-36].

### Data analysis

One-way ANOVA was used to compare timing of developmental landmarks as well as circulating hormone levels, sexual behavior and estrous cycle length. Two-way ANOVA with time as the within subjects measure and group as the between subject variable followed, where appropriate, by Tukey’s post hoc test were used to assess change in body weight, body length, anogenital index, glycemia and open field behavior. Fisher’s exact test was employed to compare presence of urine and fecal boli on the open field between groups. Statistical significance was set at *p* < 0.05 for all data. All statistical analysis was performed using SPSS (IBM, SPSS Statistics 15).

## Results

### Experiment 1: Physical development and behavior in the open field

#### Glycemic development

**Figure 2:**
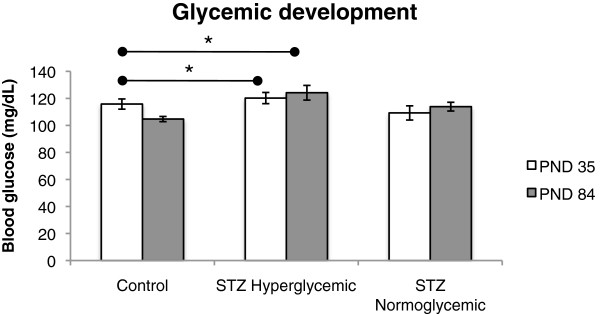
**Glycemia on PND 35 and 84 of Control (n = 10), STZ hyperglycemic (n = 6), and STZ normoglycemic (n = 6) rats.** Values expressed as mean ± standard error of mean. Significant group effect, p < .05, and trend towards significant time and group interaction, p = 0.053. *p < .05, statistically significant difference compared with Control group (ANOVA followed by Tukey’s test).

The average glycemic levels of the control group on Day 120 was 105.40±2.65 compared to 130.34±1.80 for the STZ-treated group that met the hyperglycemia criterion and 110.93±1.57 for STZ group that did not meet that criterion. Figure 2 shows mean blood glucose levels for each of these groups on PNDs 35 and 84. Overall rats in the STZ hyperglycemic group, as defined on PND 120, also had higher blood glucose levels than the control group at these earlier time points (significant main effect of group F(2,19) = 4.475, p < .05; post hoc Tukey’s test Control vs STZ hyperglycemic, p < .05). There was no overall effect of time although the time by group interaction approached significance (F(2,19) = 3.44, p = 0.053) reflecting the fact that the difference in blood glucose levels between rats in the STZ hyperglycemic group and the other two groups tended to increase over time.

#### Physical development

There were no significant differences between Control and STZ groups on any of the developmental landmarks measured (Hair appearance (Control, 6.54±0.25; STZ, 7.20±0.37, F(1,14) = 0.008, p > .05); Pinna unfolding (Control, 3.03±0.18; STZ, 3.24±0.23, F(1,14) = 0.533, p > .05); Incisor eruption (Control, 8.83±0.21; STZ, 9.32±0.36, F(1,14) = 0.330, p > .05); Eye opening (Control, 14.60±0.33; STZ, 14.34±0.22, F(1,14) = <0.001, p > .05); and vaginal opening (Control, 35.85±0.84; STZ, 35.87±0.93, F(1,14) = 1.575, p > .05).

**Figure 3:**
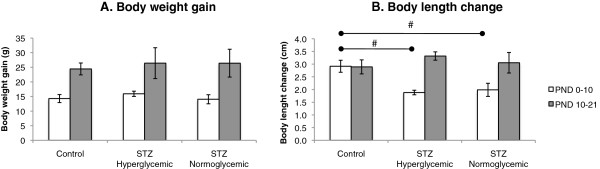
**Body weight gain and length growth between PND 0 to 21.** Body weight gain (g) **(A)** and body length growth (cm) of Control (n = 10), STZ hyperglycemic (n = 6), and STZ normoglycemic (n = 6) rats. **(B)** Values expressed as mean ± standard error of mean. **(A)** and **(B)**. Significant time effect, p < .001. **(B)** # trend toward significance of a time and group interaction effect, p = 0.064 (ANOVA followed by Tukey’s test).

**Figure 4:**
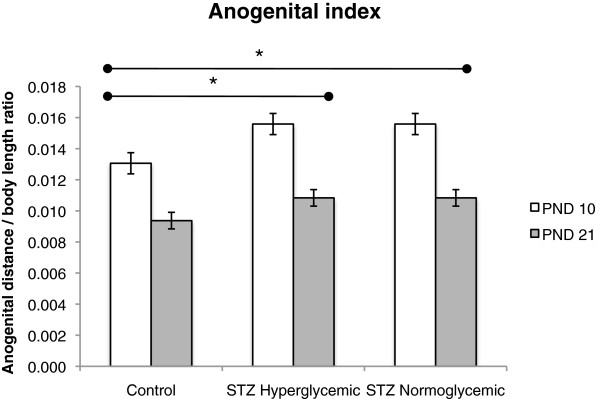
**Anogenital index of Control (n = 10), STZ hyperglycemic (n = 6), and STZ normoglycemic (n = 6) rats.** Values expressed as mean ± standard error of mean. Significant time effect, p < .0001, significant group effect, p < .05, and significant group and time interaction, p < .05. *p < 0.05 – statistically significant difference compared to Control group (ANOVA followed by Tukey’s test).

As expected, body length, body weight, and anogenital distance index of pups in all groups increased over time (F(1,19) = 9.74, p < .01, F(1,19) = 19.52, p < .001, F(1,19) = 387.06, p < .001, respectively). There were no significant differences between Control and STZ groups on body weight gain (Figure 3A). Both groups of STZ-treated pups showed a smaller increase in body length between PND 0 and 10 than pups in the Control group but all groups grew at similar rates between PND 10 and 20 resulting in a trend toward a time and group interaction effect (F(2,19) = 3.198, p = 0.064) (Figure 3B). Both groups of STZ treated rats had a higher anogenital index than rats in the control group (F(2,19) = 4.73, p < .05; post hoc tukey test control vs STZ hyperglycemic, p < .05, STZ hyperglycemic vs STZ normoglycemic, p < .05) probably due to the decreased body length of STZ rats (Figure 4).

#### Open field behavior

**Figure 5:**
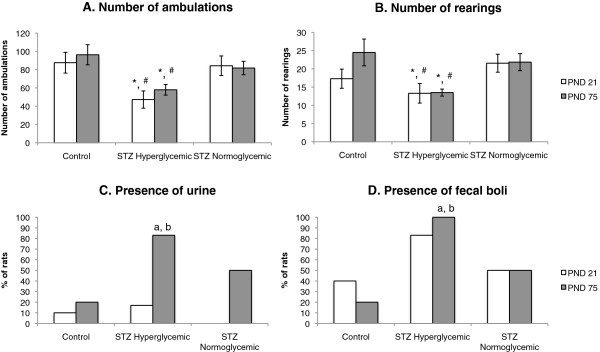
**Open field behavior on PND 21 and 75.** Number of ambulations **(A)**, number of rearings **(B)**, presence of urine **(C)** and presence of fecal boli **(D)** of Control (n = 10), STZ hyperglycemic (n = 6), and STZ normoglycemic (n = 6) rats. Figures A and B: Values expressed as mean ± standard error of mean. Figures C and D: values expressed as % of rats. **(A)** and **(B)** Significant group effect, p < .05. *p < 0.05 – statistically significant difference compared to Control group (ANOVA followed by Tukey’s test). #p < 0.05 – statistically significant difference compared to STZ Normoglycemic group (ANOVA followed by Tukey’s test). **(C)** and **(D)**^a^p < 0.05 - statistically significant difference compared to Control group (Fisher’s exact test). ^b^p < 0.05 - statistically significant difference compared to STZ Normoglycemic group (Fisher’s exact test).

Behavior in the open field for all groups on PND 21 and 75 is shown in Figure 5. Rats in the STZ hyperglycemic group had a lower ambulation score than either of the other two groups (Group effect, F(2,19) = 16.388, p < .05; post hoc Tukey test Control vs STZ hyperglycemic, p < .05, STZ hyperglycemic vs STZ normoglycemic, p < .05). Similarly, frequency of rearing was lower in the STZ hyperglycemic group than in the other groups (Group effect, F(2,19) = 6.115, p < .05; post hoc tukey test control vs STZ hyperglycemic, p < .05, STZ hyperglycemic vs STZ normoglycemic, p < .05). On PND 75 rats in the STZ hyperglycemic had a higher rate of urination and increased presence of fecal boli than rats in the other groups (Fisher’s exact test, p < .05).

### Experiment 2: Estrous cyclicity, sexual behavior and circulating gonadotropin, ovarian steroid, and insulin levels

**Figure 6:**
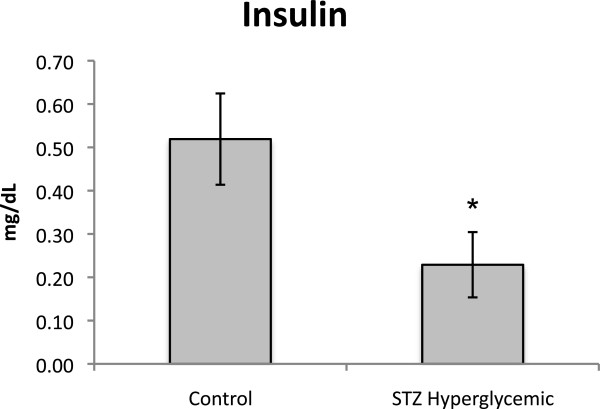
**Insulin levels on PND 84 from Control (n = 10) and STZ hyperglycemic (n = 6) rats.** Values expressed as mean ± standard error of mean. *p < 0.05 – statistically significant difference compared to Control group (One way ANOVA).

On PND 120 blood glucose levels of controls were 105,64±2.67 and of STZ-treated rats 132.30±2.20 (F(1,10) = 59.416, p < .05). In addition, STZ hyperglycemic rats had lower circulating insulin levels than Control rats on PND 84 (F(1,10) = 5.012, p < .05) (Figure 6).

**Figure 7:**
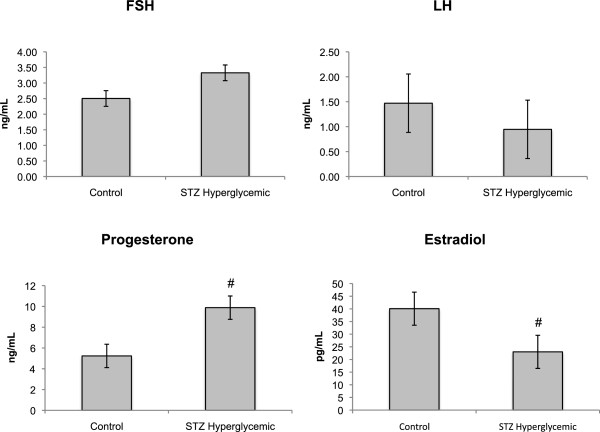
**Sexual hormones levels of Control (n = 10) and STZ hyperglycemic (n = 6) rats.** Values expressed as mean ± standard error of mean. # p < 0.10 compared to Control group (ANOVA).

There were no significant differences in estrous cycle duration (Control, 4.10±0.16; STZ, 4.62±0.31, F(1,10) = 2.281, p > .05), lordosis quotient (Control, 90.00±4.28; STZ, 86.39±3.37, F(1,10) = 0.439, p > .05), and LH and FSH levels (F(1,9) = 0.670, p > .05; F(1,9) = 1.253, p > .05 respectively) between the groups. However, as Figure 7 shows, STZ hyperglycemic rats tended to have higher serum progesterone (F(1,9) = 3.878, p = 0.08) and lower beta-estradiol levels (F(1,9) = 4.722, p = 0.058) than rats in the Control group.

## Discussion

In this study we investigated the effects of neonatal STZ injection on a broad array of developmental and adult measures in female rats. We observed acute effects of STZ injection on growth and long-term consequences of this treatment on open field behavior and on some measures of reproductive hormones.

Hyperglycemia only appeared in a subset of the STZ treated rats and did so only at PND 84. Both of these effects are consistent with earlier reports (Kiss et al., 2009). When STZ is given at such an early age the pancreatic beta-cells are partially restored and recovery rate as well as degree of recovery varies between animals. Using a similar neonatal STZ injection model, Bonner-Weir and colleagues [1] reported that STZ-treated rats were normoglycemic until 6 weeks of life. As in the current study Bonner-Weir and colleagues also found that the prolonged hyperglycemia of adult STZ rats was associated with a decrease in insulin production.

The reduced growth of STZ animals in both normoglycemic and hyperglycemic groups from Days 1–10 most probably reflects the hyperglycemia/hypoinsulinemia induced by STZ injection. Bonner-Weir and colleagues [1] found that rat pups injected with STZ on Day 2 postpartum showed a peak of hyperglycemia and a marked reduction of beta-cell numbers on Day 4 postpartum. By 10 days of age the STZ injected animals became normoglycemic with partial recovery of the beta-cell number. As a reduction in beta cell number leads to hypoinsulemia and insulin is directed related to growth [37,38], one would expect a reduction in growth during the hypoinsulemic period. However, since between PND 10 and 21 normoglycemia was already re-established with beta-cell number and insulin production partially recovered, growth rate was not impaired. A change in response to insulin, while not explaining the initial growth retardation, could contribute to the normal growth rate later in life in the face of lower insulin levels.

In the current study, rats in the STZ hyperglycemic group exhibited diminished locomotor and exploratory behaviors in adulthood compared to the control group and this difference was associated with increased urination and presence of fecal boli. Both of these measures are often used as indices of anxiety and suggest higher anxiety in the STZ hyperglycemic group than in controls. Other authors using models similar to that used here have reported these effects only in infancy [39] but reductions in locomotor and exploratory behaviors are commonly observed in adulthood in severely diabetic rats. For example, Grzeda et al. reported decreased locomotor and exploratory activity in the open field in diabetic rats [12]. In addition, several studies of severely diabetic rats report a reduction in the frequency of crossings and rearings in the open field [11-15]. In another study, Bellush and colleagues [11] demonstrated that diabetic rats showed decreased locomotion only on the first exposure to an open field suggesting that diabetes-induced disruptions in open-field activity are related to anxiety rather than to motor or energy deficits. In addition, Ramanathan and colleagues [13,14] showed that diabetic rats were more anxious than non-diabetic animals in the open field, elevated plus maze and social interaction test. In the current study only the hyperglycemic STZ-treated rats showed these changes in open field behavior suggesting that it was the long term consequences of this early manipulation on metabolism rather than the early manipulation *per se* that induced these behavioral changes. ln conclusion, the open field results for the mild hyperglycemic rats from the present study are in agreement with several studies using severely diabetic rats, suggesting that glycemic levels do not necessarily have to exceed 300 mg/dL to impair behavior in both infancy and adult life.

It is well established that diabetes impairs reproductive function [16,17] and embryonic development [40,41]. Results of several studies have demonstrated an impaired LH surge in severely diabetic rats [42-45] although basal levels of LH and FSH levels did not differ between diabetic and nondiabetic rats [46,47]. Consistent with the latter findings, in the current study hyperglycemia was not associated with any change in circulating gonadotropins. However, again similar to the result of studies of severely diabetic animals [48] ovarian hormone levels were altered .in hyperglycemic STZ-treated rats.

One possible explanation for this is the role of insulin in modulating ovarian steroid production. It is well-established that both insulin and IGF-I synergize with gonadotropins to enhance granulosa cell function [49,50] and lack of insulin may interfere with the action of gonadotropins on granulosa cells [47] and impair the synthesis of ovarian hormones (see [51], for a general review in mammals). Such a mechanism would not however, explain the higher levels of progesterone and lower levels of beta-estradiol in the STZ rats of the present study. An alternative mechanism in which diabetes either inhibits the conversion of progesterone to testosterone or the aromatization of testosterone to estradiol has been suggested by Meurer and colleagues [47] and such a mechanism would explain the current data.

In addition to hormonal changes, severely diabetic rats present other reproductive deficits including alterations in the estrous cycle such as anovulation [48,52] and increases in cycle length [53]. Lower lordosis quotients in sexual behavior tests have also been reported [18,19]. Estrous cycle duration and sexual behavior were not impaired in the STZ rats in the present study. The fact that mild hyperglycemic rats from the present study did not show the same reproductive impairment as severe diabetic rats reinforces the idea that the impairment of reproductive function is dependent on the intensity of hyperglycemia.

In the current study, STZ injection in the neonatal period caused hyperglycemia/hypoinsulinemia in adult life. Lack of insulin on the first days following STZ injection probably contributed to reduced growth rate, and the re-establishment of normal growth most likely reflected the partially recovery of insulin production. Hyperglycemic STZ-treated rats exhibited diminished locomotor and exploratory behavior in the open field in adulthood. Mild hyperglycemia did not impair gonadotropin levels although ovarian steroid hormone levels were altered. The changes in general activity and hormone profile observed in mild hyperglycemia are similar to those shown in studies of severely diabetic animals. Overall these data suggest that even mild hyperglycemia/hypoinsulinemia is sufficient to impair general activity and sexual steroid hormone secretion in female Wistar rats.

## Competing interests

The authors declare that they have no competing interests.

## Authors’ contributions

ACIK participated in the conception and design of the study, performed most of data acquisition (mating, vaginal smears, streptozotocin administration, blood samples for glycemia and hormonal determinations, physical development, open field behavior, estrous cycle, and sexual behavior) interpreted the data, performed the statistical analysis, prepared the figures and drafted the manuscript. BW helped drafting the figures, statistical analysis, advising the best test to be performed for each parameter, and has been deeply involved in drafting the manuscript and revising it critically. YKS participated in the design of the study, was deeply involved in acquisition and interpretation of data, especially regarding STZ administration, blood samples, and insulin determination by ELISA, and helped to draft the methods, results and discussion section of the manuscript regarding this data. MMB participated in the conception and design of the study, especially regarding the methods to study physical development and open field behavior, participated on the interpretation of data, and helped to draft the methods, results and discussion section of the manuscript regarding this data. WDGK participated in the conception and design of the study, especially regarding the methods to study sexual behavior and estrous cycle, participated on the interpretation of data, and helped to draft the methods, results and discussion section of the manuscript regarding this data. JAAF participated in the conception and design of the study, especially regarding the methods to measure circulating gonadotropin and ovarian steroid levels, performed the hormonal determinations, participated on the interpretation of hormonal data, and helped to draft the methods, results and discussion section of the manuscript regarding this data. DCD conceived of the study, and participated in its design and coordination and helped to draft the manuscript. All authors read and approved the final manuscript.
